# Investigating the Effectiveness of a Virtual-Reality-Based Mindfulness Intervention on Internet Gaming Disorder

**DOI:** 10.3390/bs14121137

**Published:** 2024-11-27

**Authors:** Selma Tvrtković-Hasandić, Pınar Ünal-Aydın

**Affiliations:** Department of Psychology, International University of Sarajevo, 71000 Sarajevo, Bosnia and Herzegovina; paydin@ius.edu.ba

**Keywords:** internet gaming disorder, mindfulness, virtual reality

## Abstract

Novel treatment approaches for Internet Gaming Disorder (IGD) include the use of mindfulness and technology-based interventions. Mindfulness has been shown as a protective factor against IGD, but the treatment dropout rates are high due to long sessions and treatment duration. Pathological gamers show approach bias towards technological gadgets, and the inclusion of Virtual Reality has been effective in IGD treatment. Due to the effectiveness of mindfulness and the attractiveness of VR, a combined intervention could decrease treatment time and willingness for treatment. Therefore, the aim of this study was to examine the effectiveness of a brief VR-based mindfulness intervention on IGD symptomatology. Nine participants meeting the IGD symptom criteria, ten recreational game users, and eight healthy controls without a gaming history participated in the study. The intervention consisted of four weekly 20-min-long Attentional Focus Mindfulness sessions. The results indicate a significant reduction in IGD symptoms and weekend gaming time in the treatment group. Despite the small sample size in the treatment group (n = 9) and lack of randomization, the findings constitute a valuable starting point. As a cost- and time-effective intervention, this approach could reduce dropout rates and increase treatment adherence, especially in younger gamers. Additional studies with a larger sample size, randomization, and a longitudinal approach are needed to further validate the found results.

## 1. Introduction

The rates of addictive behaviors have been increasing in recent years, particularly for nonsubstance-related addictions [1]. Internet-related activities, such as Internet gaming, are especially concerning due to the easy access to the Internet [2,3]. The latest gaming data report approximately 3 billion active gamers worldwide [4]. Due to an increase in the number of gamers and its associated negative consequences, the American Psychiatric Association (APA) has included Internet Gaming Disorder (IGD) in the appendix of the latest edition of the Diagnostic and Statistical Manual of Mental Disorders (DSM-5) under nonsubstance addictions [5]. The symptoms include a preoccupation with gaming, withdrawal and tolerance, loss of control and interest in other activities, continuation with gaming despite negative consequences, deception, and the use of games to escape or relieve negative moods. To be diagnosed with IGD, one must meet at least five symptoms within the past year [5]. The total number of gamers with IGD is unclear. Some studies estimate a 4–12% prevalence of IGD among adolescents and adults [6]. Other studies claim that the majority of gamers do not experience any IGD symptoms, and that the percentage of those who might is extremely low (i.e., 0.3–1.0% of the general population might qualify for a potential diagnosis) [7,8,9].

A key discriminatory factor between passionate gaming and pathology is the experience of distress [7,8,9]. The most prominent reason for gaming is the wish to unwind, relax, and decompress (reported by 66% or 2.04 billion gamers) [4,10]. While gaming is most frequently used to unwind, for some people, this develops into a maladaptive pattern of behavior [11,12,13]. For example, due to the temporary escape into a virtual world, individuals may use gaming as a form of maladaptive coping to manage real-life problems and stress [6,14,15]. This form of behavior (i.e., “escapism”) is a common trigger and behavioral pattern among addictive behaviors [16]. Among pathological gamers, escapism to avoid feelings of stress is the most common factor (present in 80% of cases) [17]. In addition to being a trigger, escapism is also a maintenance and relapse factor [14]. In the long run, it may lead to more symptoms and psychological distress (e.g., more stress, depression, and anxiety) [16,18,19] and to more gaming to relieve these negative states [20,21,22].

Currently, there is no uniform treatment for IGD. This is mostly due to the lack of universally established IGD assessment criteria and assessment tools [23,24]. Research shows that a combination of different treatments is usually the most effective in dealing with IGD symptomatology [23,24,25]. Under the most frequently used treatment options are classical pharmacotherapy and CBT-based psychotherapy [24]. In addition, there has been a shift to newer and adjusted treatment methods, such as various mindfulness-based interventions [24,26] and, more recently, technology-based treatment approaches [25].

Mindfulness enables a “being-in-the-moment” experience [6]. Practicing mindfulness helps with self-management of negative emotions, attentional switching, and attentional regulation [27]. These factors have been evidenced as particularly important in substance use disorders [28], making mindfulness a promising intervention for treating addictions and addiction-related behaviors [6,29]. By leading to the self-regulation of attention (i.e., being present in the moment without judgment or elaboration, and awareness and acceptance of one’s thoughts, feelings, and sensations) [30], mindfulness impacts the reward system. Addictive behaviors are characterized by increased attention regarding short-term goals and lost sight of long-term objectives. Mindfulness helps to bring back awareness to long-term objectives [31]. By focusing on acceptance and non-judgment regarding current experiences, these interventions lead to behavioral change—a key aspect of any psychological treatment [27]. In addition, mindfulness also reduces symptoms of stress, anxiety, and depression, and it increases relaxation and pleasant affect [32,33,34,35]. In relation to IGD, previous studies have shown that mindfulness is negatively associated with Internet gaming behaviors, serves as a protective factor [2,26,36], and is an effective treatment approach for IGD [6].

Quite recently, the interest in the use of tech-based tools for assessing, preventing, and treating IGD symptoms has increased [25,37,38,39]. Technology has become irreplaceable in today’s world. This is especially true for the younger generations, which makes it an appealing tool for treatment interventions [25]. Such treatments have been demonstrated to be effective in mood disorders, gambling disorder, and substance addictions, suggesting them as a promising tool for IGD treatment as well [25,40]. One of the most popular tools is Virtual Reality (VR) [25].

The primary application of VR in addictive disorders has been to reduce cravings by subjecting patients to stimuli associated with the addiction [41]. It has been used in a similar manner in IGD [42,43], and such interventions have been shown to successfully reduce gaming symptoms similarly to CBT [38,39,44]. VR is generally used as a complementary approach to the conventional treatment methods [45] as a stimulus to elicit and tackle cravings and for recreating a real-life experience, motor behaviors, and for stimulus exposure [42,43]. Its ability to create a flowing, immersive, and interactive environment [46], combined with the general appeal of modern technology, make VR especially attractive for young people and can increase motivation regarding mental health treatments [47]. The use of VR in interventions provides a technological outlet during treatment. For the most effective outcome, the treatment of behavioral addictions usually aims at moderate engagement in the behavior rather than complete abstinence [48]. Additionally, individuals with IGD symptoms show automatic approach behaviors to anything related to gaming. VR can elicit such an approach, further motivating research regarding similar treatment interventions [49].

The motivation and approach behaviors towards VR may be explained via Self-Determination Theory (SDT) [50].

SDT is a theory of human motivation and psychological functioning, which assumes the presence of three innate psychological needs: autonomy (a desire for freedom of choice and a sense of control), competence (a sense of mastery), and relatedness (a sense of connectedness). Humans are driven by intrinsic or extrinsic motivation to fulfil these needs [50]. When people are intrinsically motivated, they perform an activity due to enjoyment and inner satisfaction, while extrinsic motivation aims at achieving an instrumental value [50].

For intrinsically motivated individuals, the entire process is the goal of the activity [50], and, the more enjoyable a task is, the more intrinsically motivated an individual becomes. Facilitating motivation that is autonomous is crucial for successful behavioral intervention as it increases adherence [50].

SDT can be used to explain the motivational pull of video games [51]. Gamers are usually intrinsically motivated towards gaming. Playing games satisfies the needs for autonomy by availing various possibilities in the gaming world, competence by achieving success in games, and connectedness via gaming characters and social interactions [52,53,54]. The successful satisfaction of these needs usually leads to increases in mood and motivation for future play [54,55,56,57]. Virtual environments can also fulfill all these needs, especially autonomy and competence [58,59]. A study by Reer et al. [58] found that the use of VR shapes the gaming experience even more by providing a more natural setting, increases the enjoyment of the experience [60,61], and involves presence and positive affect [60]. The motivational pull of VR can be additionally highlighted via the concepts of immersion and presence [62,63]. Immersion is a state of high cognitive involvement, while presence is the feeling of being in the VR rather than the real word [62,63]. As key affordances of VR, they have been shown to increase perceived autonomy and competence [64].

Research has shown that SDT can be applied in psychotherapy [65]. For intrinsically motivated users, VR can be used as a tool to increase behavioral intention for an activity [66], as a supplement to enhance interest in service use [67,68,69], and it reduces the importance of short-term objectives [70].

### 1.1. Limitations to Current Treatment Approaches

The classical treatment approaches lack a consensus regarding the treatment guidelines, resulting in uncertain and inconsistent treatments [71]. For example, the treatment of choice for IGD is CBT. Multiple variations of CBT are used to treat IGD, and there is a lack of concrete and standardized session guidelines for an optimal intervention [72]. Additionally, the effectiveness of CBT has been shown to depend on the patient profile, with more effective treatment outcomes in older rather than younger populations (i.e., adults compared to adolescents). This could be due to the unwillingness of adolescents to share personal details in sessions, especially with adults, the presence of shame and stigma [73], and the general lack of willingness regarding treatment participation. Adolescents mostly believe that they can manage problems on their own, or with the help of non-professionals, such as friends [74,75]. In order to increase the effectiveness among adolescent patients, the research shows that an adaptive, supportive, and structured environment is needed [76,77].

The limitations in the current IGD treatment studies are the nature of the control groups and gender inclusion [78]. A recent study [78] has highlighted a lack of non-gamers as control groups in the IGD literature and has recommended the use of two control groups: both recreational gamers and non-gamers. Only including gamers limits the generalizability of the data, while using non-gamers may create some limitations in terms of the validity of the measures. In addition, there is a lack of mixed-gender research. According to the latest reports, 53% of gamers (approximately 1.7 billion) are male, while 47% are female (approximately 1.4 billion) [10,49,78]. The literature shows that the number of people suffering from IGD has increased significantly in the last decade [2], and so has the number of female gamers (i.e., the ratio in 2006 was 62% to 38%) [10]. Unfortunately, most of the research, including treatment studies, includes male participants only. There is a rising importance of involving female gamers in treatment research [78].

### 1.2. The Present Study

Based on the review of the previous treatment studies and relevant theories, we believe that there is a lack of a standardized IGD treatment that is both attractive and efficient.

CBT has demonstrated effectiveness in IGD treatment, but it is difficult to create uniform session guidelines [72]. In addition, it may carry stigma and reduce treatment willingness in younger patients [73]. Mindfulness interventions can be time-consuming and long, often resulting in patients dropping out in the middle of the treatment. The average attendance of mindfulness interventions is four, with the number of dropouts increasing thereafter [79].

Recently, brief mindfulness interventions have been proven to be just as effective as long-format sessions. When compared, 4- and 8-week mindfulness interventions show no difference in their effectiveness regarding symptom reduction [80], and shorter session durations (e.g., a 20-min-long FA mindfulness intervention) have also demonstrated effectiveness [81].

The previous findings report that brief and shorter-duration mindfulness sessions are considered to be more beneficial in IGD, especially for younger individuals [79,80,81,82,83,84,85,86,87].

Compared to the traditional treatment methods, the approach used in this study aims at minimizing therapist and session errors and increasing intervention consistency by utilizing a standardized VR platform available to all practitioners. In addition, it has the potential of reducing stigma and increasing willingness for treatment by taking advantage of the motivational pull of VR.

To our knowledge, no study has utilized VR as a method to implement mindfulness interventions in a gaming population. Classic mindfulness and VR-cue exposure interventions have been successful in IGD treatment. If a combination of these two approaches were to be demonstrated as effective, it could potentially reduce stigma, treatment cost, time, and increase adherence. The aim of our study is to investigate the effectiveness of a VR-based mindfulness intervention in IGD. Based on previous studies, we hypothesize that a 4-week VR-based mindfulness intervention will be successful in reducing IGD symptomatology, and that this change will be significantly associated with gaming patterns and the variables of stress, positive affect, and negative affect.

## 2. Materials and Methods

### 2.1. Participants

This study included one treatment group with participants who met at least one IGD diagnostic criterion and two control groups: recreational gamers and non-gamers. In addition, the study included both male and female participants in all three groups. The total study sample consisted of 27 participants. The treatment group initially consisted of eleven participants meeting 1–5 DMS-5 criteria for IGD. Out of them, one participant met 5 IGD diagnostic criteria, while two participants met 4, two participants met 3, two participants met 2, and four participants 1 diagnostic criterion. During the intervention period, one participant completed the VR sessions but was unwilling to fill out the forms post-test, and one participant dropped out after one session, leaving the total IGD sample at nine participants. The recreation game user group consisted of ten recreational gamers who did not meet any IGD diagnostic criteria and eight healthy control participants who do not game at all. Inclusion criteria for the IGD group included having at least one IGD diagnostic criterion and actively playing video games. Control subjects either played video games but did not meet any IGD diagnostic criteria (recreational game users) or have never played video games (healthy controls). All participants were of legal age, fluent in English, and were free from a current psychiatric diagnosis and medication use, and had no cognitive, auditory, or vision impairments. Ethical approval for this study was obtained by the ethical council of the International University of Sarajevo (IUS-REC-01-3528/2023).

### 2.2. Instruments

#### 2.2.1. Sociodemographic Form

A sociodemographic form included age, gender, faculty, study year, psychiatric disorder diagnosis, weekly gaming time, weekend gaming time, daily time spent online, gaming device, and game type.

#### 2.2.2. Ten-Item Internet Gaming Disorder Test (IGDT-10; [88,89])

The Ten-Item Internet Gaming Disorder Scale (IGDT-10; ref. [89] was developed by Kiraly et al. in 2019 as a short screening instrument to assess IGD according to its proposed DMS-5 criteria. It includes ten items, each reflecting one of the diagnostic criteria for IGD. Items nine and ten represent the same dimension and are evaluated as one symptom (the response “often” on either one or both equals to one point in the scoring). Considering the presence of symptoms over the past 12 months, the IGDT-10 is a 3-point-Likert scale (i.e., “never”, “sometimes”, or “often”), where a score of 5 and above shows a clinical presence of IGD (higher scores indicate higher symptom severity). To reflect the dichotomous structure of the DSM-5 criteria, the responses “never” and “sometimes” are evaluated as criterion not being met (0 points), while the response “often” is evaluated as criterion met (1 point). Examples of the items include “Have you ever unsuccessfully tried to reduce the time spent on gaming?” and “When you were not playing, how often have you fantasized about gaming, thought of previous gaming sessions, and/or anticipated the next game?”. In this study, Cronbach’s α was 0.68.

#### 2.2.3. Perceived Stress Scale (PSS-10; [90])

The Perceived Stress Scale (PSS-10; [90]) is a 10-item self-report scale used to measure the degree to which current life situations are appraised as stressful. Developed by Cohen et al. [90] in 1983, PSS is 4-point Likert scale (0 = never to 4 = very often) assessing perceived stress (i.e., helplessness and lack of self-efficacy) over the past month. Higher scores indicate higher levels of perceived stress. Exemplar items are “In the last month, how often have you been able to control irritations in your life?” and “In the last month, how often have you felt that you were on top of things?”. Scores ranging from 0 to 13 would be considered low stress, from 14 to 26 moderate stress, while scores ranging from 27 to 40 would be considered high perceived stress. Cronbach’s α in this study was 0.84.

#### 2.2.4. The Positive and Negative Affect Schedule-Short Form (PANAS-SF; [91])

The Positive and Negative Affect Schedule-Short Form [91] is a self-reported five-point Likert scale measuring positive affect (PA) and negative affect (NA). Individuals report how they have felt in terms of their affective states (e.g., “Guilty”; “Interested”) in the past seven days, ranging from “very slightly or not at all” to “extremely”. Developed by Watson et al. in 1988, the scale includes 20 item words, where 10 of them represent positive affect (e.g., “Interested”) and ten represent negative affect (e.g., “Guilty”). Scores range from 10 to 50 on each dimension, where higher scores indicate higher affect (both positive and negative). In this study, Cronbach’s α study was 0.70.

#### 2.2.5. The Internet Addiction Test (IAT; [92])

The Internet Addiction Test [92] is a self-reported 20 item, 5-point Likert scale that is used to assess addictive patterns of Internet use. The scale developed by Young in 1998 has a maximum score of 100, with a higher score indicating higher severity of Internet addiction. A score of 30 and below is considered a normal level of Internet use, 31–49 indicates the presence of a mild level of Internet addiction, 50–79 reflects the presence of a moderate level, and scores ranging from 80 to 100 indicate a severe Internet addiction. In this study, Cronbach’s α was 0.92.

#### 2.2.6. Amelia Virtual Care [93]

Amelia Virtual Care (formerly known as Psious; [93]) is a VR platform used for mental health interventions. It was founded in 2013 by Xavier Palomer in Barcelona, Spain. The software was initially developed in 2013, with Sessions are conducted using VR goggles and environments (e.g., public speaking, high places, flying, tight spaces, moods, eating, etc.). The mindfulness-based interventions include Attentional Focus, body scanning, emotional regulation, and underwater in the ocean.

The Attentional Focus environment was used in this study. This virtual environment allows participants to “practice mindfulness and acceptance exercises as they walk through a peaceful meadow”. Based on the concept of Vipassana meditation, it features the major aspects of mindfulness and trains the capacity to remain in the “here and now”. This 20 min practice encourages patients to minimize their internal noise and to distance themselves from their thoughts by letting unwanted thoughts in and welcoming them without judgment or reaction [94].

### 2.3. Study Procedure

Students in Bosnia and Herzegovina (i.e., Kanton Sarajevo) were scanned via Google Forms for their gaming patterns (i.e., IGD symptoms, weekly, and weekend gaming time). The online survey used for screening included an explanation about the study procedure and aims, and participants had a chance to leave their contact information (i.e., phone number) if interested in further participation. Volunteers who met the inclusion criteria were contacted for further study participation. Those who were eligible for the treatment group started VR-based mindfulness interventions from May 2024 until June 2024. Depending on their gaming patterns, others were contacted to be part of the control groups.

Considering the limitations and suggestions of previous studies, this study included two control groups (i.e., recreational gamers and non-gamers) and both male and female participants [78].

At baseline, all participants completed self-report scales on IGD symptoms (IGDT-10), gaming time (weekday and weekend), gaming device and type of game played, daily online time, stress (PSS), and positive and negative affect (PANAS-SF). In addition, the intervention group completed a self-report scale on Internet addiction (IAT).

Based on previous findings, a 4-week, 20-min-long VR-based mindfulness intervention (i.e., AF mindfulness; [81]) was utilized for this study.

The VR sessions were held once a week in a quiet university lab setting on the same day as the previous sessions (e.g., each Wednesday). To ensure consistency of the intervention, all participants completed the sessions in the same room, were exposed to the same environment (Attentional Focus; [94]), and used the same earbuds, laptop (HP), and headset (Pico G2). The lab was placed in a quiet area of the university, safe from outside noises that could interrupt the sessions. The VR software (XRHealth Portal 4.0.17) in this study featured a voice-guided mindfulness exercise and provided participants with all necessary information about the session flow. Therefore, all participants received the exact same input. No prior training was required to conduct the sessions since the session guidelines were provided by the Amelia VR software (XRHealth Portal 4.0.17). To protect anonymity, all participants received a user ID composed of numbers and letters that was used to log in during their weekly sessions.

The session duration was 20 min (80 min in total). The environment was set in the countryside and featured a peaceful walk through a meadow, including changing views of trees and falling leaves, flowers, and the sounds of a nearby river, rustling leaves, and flaps of butterflies [94]. During the session, participants were asked to direct their focus on different stimuli within the VR environment, to try to remain in the moment, and to accept any feelings or thoughts arising during the intervention. The participants were not talking or talked to during the duration of the session, and there were no outside noises that would disrupt the session flow.

After the interventions were completed (for the treatment group) or 4 weeks passed since the baseline screening (for the control groups), the participants self-reported their gaming patterns (gaming time, device, and type of game played) and filled out the same study scales as at baseline (i.e., IGDT-10, PSS, PANAS-SF, and IAT).

### 2.4. Statistical Analysis

At baseline, demographic characteristics and scale scores were compared between the three groups using chi-squared test for gender, gaming device, and type of game played and Independent-Samples Kruskal–Wallis test for age, gaming time, daily online time, and scores on IGDT-10, PSS, and PANAS-SF.

To measure treatment efficacy on IGD symptoms, repeated measures ANOVA was used with a within-subjects factor of time (i.e., pre-test–post-test) and a between-subjects factor of group (i.e., treatment and control groups). Even though the data have a non-normal distribution, a repeated measures ANOVA has been proven to be robust to non-normality [95] and has been used in clinical studies as there is no non-parametric equivalent and some data cannot be transformed to meet the normality assumption [96,97].

Following the recommendations from previous studies on IGD treatment [98] and to further aid the findings from the repeated measures ANOVA, an additional analysis in the IGD group included a Wilcoxon signed rank test to measure changes in IGD symptoms and gaming time during weekdays and weekends. Spearman correlations were then used to show relationships between the changes in mean values of the study variables in the IGD group [98].

## 3. Results

### 3.1. Baseline Characteristics of the Sample

No significant differences were found between the groups in age (z = 3.96, *p* = 0.138), daily time spent online (z = 3.73, *p* = 0.155), PSS (z = 2.92, *p* = 0.233), and PANAS-P scores (z = 0.99, *p* = 0.608; Table 1).

As expected, significant differences between the groups were found in IGD scores (z = 24.74; *p* < 0.001), weekday (z = 16.33, *p* < 0.001), and weekend gaming time (z = 21.66, *p* < 0.001; Table 1). The pairwise comparison for IGD scores reported no significant difference between the two control groups (z = 0.00, *p* = 1.00). A significant difference was found between both control groups and the treatment group (z = −13.50, *p* = 0.000).

For weekday gaming time, the pairwise comparison showed that the difference was not significant between the control groups (i.e., recreational game users and healthy controls; z = 3.14, *p* = 1.00), while the differences between the HCs and IGD group (z = −14.44, *p* < 0.001) and HCs and RGUs (z = −11.30, *p* = 0.002) were significant. Gaming time during weekends showed a significant difference between the two control groups (z = −0.95, *p* = 0.020), HCs, and IGDs (z = −17.44, *p* = 0.000) and no significant difference between the RGUs and IGDs (z = −7.49, Bonferroni corrected *p* = 0.104).

In PANAS-N, there was a significant difference between the groups (z = 7.78, *p* = 0.020). The pairwise comparison shows that this difference was not significant between the control groups (z = −2.51, Bonferroni adjusted *p* = 1.00) and the RGUs and IGDs (z = −7.63, Bonferroni adjusted *p* = 0.108), while a significant difference was found between the HCs and IGDs (z = −10.15, with Bonferroni adjusted *p* = 0.025).

There were no significant gender differences between the groups (z = 0.93, *p* = 0.336), nor within the RGU (z = 0.40, *p* = 5.27) or IGD group (z = 1.0, *p* = 0.317).

The type of game played showed significant differences between the groups (z = 21.89, *p* = 0.003), but this difference was not significant in the RGU (z = 2.00, *p* = 0.527) and IGD groups (z = 4.78, *p* = 0.189). There were no significant differences between the groups in terms of the gaming device used (z = 6.93, *p* = 0.074).

### 3.2. Effectiveness of the VR-Based Mindfulness Intervention

A repeated measures ANOVA showed significant main effects of time and group as well as an interaction between time and group.

A within-subjects contrast revealed that, for the control groups (both HCs and RGUs), time had no significant effect on IGD symptoms (F(1, 9) = 2.25, *p* = 0.168 for the RGU group), while, for the IGD group, this effect was significant (F(1, 8) = 7.11, *p* = 0.029).

The between-subjects effects showed no main effect of group in the control groups (F(1, 9) = 2.25, *p* = 0.168 for the RGU group) but demonstrated a significant main effect in the IGD group (F(1, 8) = 25.92, *p* < 0.001; Table 2).

### 3.3. Changes in IGD Symptoms and Weekday and Weekend Gaming Time After 4 Weeks of VR-Based Mindfulness Intervention

The Wilcoxon signed rank test showed that the VR-based mindfulness intervention elicited a statistically significant change in IGD symptoms after the 4-week intervention in the IGD group (z = −2.46, *p* = 0.014), a significant change in weekend gaming time (z = −2.12, *p* = 0.034), and no significant change in weekday gaming time (*p* = 0.334; Table 3).

### 3.4. The Correlations Between Gaming Patterns and Stress, Affect, and Internet Addiction

The Spearman correlations at post-treatment for the IGD group showed no correlation of IGD symptoms with any of the other variables (PSS, r = −0.004, *p* = 0.991; PANAS-P, r = −0.147, *p* = 0.706; PANAS-N, r = −0.211, *p* = 0.586; IAT, r = 0.621, *p* = 0.074; weekday gaming time, r = 0.573, *p* = 0.136; weekend gaming time, r = 156, *p* = 0.689; gaming device, r = 0.574, *p* = 0.106; game type, r = 0.00, *p* = 1.00).

## 4. Discussion

The present study evaluated the efficacy of a brief 4-week VR-based mindfulness intervention on IGD symptom reduction. Overall, the findings indicated that a brief, voluntary engagement in a VR-based mindfulness treatment produced a significant improvement in IGD symptoms. Although gaming time is not a direct indicator of disordered gaming [99], the IGD risk group showed a significant reduction in gaming time during the weekend compared to the baseline. These findings provide preliminary support for utilizing VR-based mindfulness interventions that are brief and short in treatment length to reduce IGD symptom severity and produce adaptive changes in gaming-related behavior. To the best of our knowledge, this is the first study that uses a VR-based mindfulness intervention for treating IGD symptoms.

The found improvement in IGD symptoms after a mindfulness intervention is in line with the previous findings [2,6,29]. The potential reasons for the treatment effectiveness include positive changes in emotion regulation, attention regulation, and the reward system [2,30,31,100,101], all directly relevant to the components of the used Attentional Focus VR environment.

In substance use disorders, mindfulness has been demonstrated as a strong mediator between emotion regulation difficulties and substance abuse [102]. A similar implication could exist for IGD. Interventions that target emotional regulation and self-control (such as mindfulness-based interventions) seem to be efficient regarding IGD treatment via reductions in urgency and premeditation [103]. Gaming is often related to emotion-driven impulsivity (e.g., feelings of urgency and premeditation), and mindfulness practices may reduce this impulsivity [2,101]. By reducing impulsivity, mindfulness might increase self-control and emotional regulation and thus reduce pathological gaming use.

Mindfulness interventions also lead to self-regulation of attention [30]. The VR environment used in this study was Attentional Focus, and its main aim is to train attention regulation. This includes accepting feelings and thoughts and allowing them in without judgement. By being able to achieve this, mindfulness impacts the reward system and reduces the importance of short-term goals while simultaneously refocusing awareness regarding long-term objectives. Addictive behaviors often result from a loss of focus on long-term objectives, and, by targeting the reward pathways, one can reduce addictive patterns of behavior [31,100].

In addition to reducing impulsiveness and increasing the focus on long-term objectives, research also shows that implementing mindfulness practices reduces stress [104]. Previous studies claim that pathological gamers tend to have higher stress levels [14,15,105,106,107,108,109]. Compared to the control groups in our study, the IGD group reported a higher stress level, even though this difference was not significant. As mentioned before, stress and the wish to escape it are the main triggers for pathological gaming [110]. For example, a study by Song and Park from 2019 [111] showed that dispositional mindfulness is a partial mediator of the association between stress and Internet addiction, and a similar implication is relevant for IGD. By targeting and reducing stress levels and escapism, we may reduce the need for pathological gaming and its symptomatology.

An unexpected finding in our study is that, regarding the pre- and post-test measures, the IGD group showed no correlation between stress and IGD symptoms. These findings may be explained by the fact that all three groups in our study reported a “moderate stress” experience. It is possible that the stress level experienced by the IGD group during the study time was not high enough to be the causal factor for pathological gaming. In addition, the scale used to measure IGD considered only the presence of severe symptoms. The scale scoring could possibly have influenced their association.

Gaming can also have positive effects, such as reductions in stress [112] and anxious and depressed states [113] and increases in problem-solving skills and wellbeing [114], optimism, and motivation [115]. The previous research shows a causal relationship between playing online games and positive emotions [57], and it is often used as a therapeutic tool in treating depression and anxiety [116]. Due to these positive outcomes of gaming, it could be possible that the treatment group in our study benefited from games in terms of stress reduction and good problem-solving skills, which possibly helped them to deal with the demands in their everyday lives.

Additionally, there was no significant difference between the groups in terms of positive affect, with the IGD group having a higher level of positive affect than the healthy controls. It could be that positive affect may have dampened the level of perceived stress and increased the general sense of wellbeing in the sample of pathological gamers. Positive affect has been shown to reduce the harmful effects of psychological stress and to serve as a buffer between stress and coping [117,118].

Even though there was a significant difference between the groups regarding negative affect, there was no significant correlation to IGD symptoms regarding the pre- and post-test measures. The presence of higher levels of positive affect may be the factor influencing the lack of a significant relationship between those two variables and IGD symptoms, which is in congruence with the previous findings [117,118].

One of the features of IGD that is up for debate in terms of its pathological qualities is gaming time [119]. Excessive gaming time is usually considered as a factor associated with gaming addiction, but, currently, there is a lack of evidence-based understanding of the relationship between time spent gaming, pathological gaming, and the severity of symptoms [120]. Additionally, in the International Classification of Diseases 11th Edition (ICD-11), the World Health Organization did not include any specifics related to gaming time in order to diagnose pathological gaming [3].

In many studies, gaming time is not included as the primary outcome regarding treatment, with the focus being solely on IGD symptomatology [6,36,121,122,123,124]. In this study, the gaming time (on both weekdays and weekends) did not differ between gamers with and without IGD symptoms. In addition, gaming time was not significantly correlated with IGD symptoms regarding either the baseline or post-test measures, which is in congruence with the previous findings [125]. A study by Cekic et al. from 2024 [125] found no correlation between IGD and playing time, and the mere time spent on gaming is usually a poor predictor of negative outcomes for gamers [126,127]. Comparing the baseline and post-test measures in the IGD group, the VR intervention did significantly reduce the gaming time during the weekend, while the gaming time during the weekdays did not show any significant change. In previous studies, it was shown that weekend gaming time (especially during the mornings) was associated with IGD symptoms, and that IGD predicted weekend gaming time [119]. Weekends are usually used to enjoy other activities and be around people, and devoting this time to gaming may be related to pathological gaming [119]. The overlap between pathological gaming and time spent gaming is larger among younger gamers, and gaming time is of greater importance to younger gamers compared to older ones [128]. Our sample consisted of university students, all over the age of 20, which may further explain the above-mentioned results, or lack thereof.

### 4.1. Strengths of the Study

The strengths of this study lie in the inclusion of a scale that measures IGD symptoms based on DSM-5 criteria rather than a modified Internet addiction scale, which is often the case [38,124,129,130], the inclusion of both recreational gamers and non-gamers as control groups, the inclusion of both male and female gamers [78], and the first-ever use of a VR-based mindfulness intervention on an IGD sample. These early findings are promising but need to be replicated in larger samples with higher symptom severity.

The additional benefits of this study relate to its simplicity and time- and cost-effectiveness. Currently, regular mindfulness interventions last approximately 8 weeks, with session durations of up to 90 min, while this approach utilizes a 4-week-long intervention with a session duration of 20 min. The dropout rates in regular interventions are high, especially in the younger population. This is an important factor to consider since the majority of IGD patients are teens and young adults [4,7,131]. In addition, this intervention may be a promising tool for younger patients and may increase treatment adherence. Combining the attractiveness of VR with the effectiveness of mindfulness for IGD treatments can create an appealing treatment approach. This would not only make it cost- and time-effective but may reduce dropout rates and significantly reduce the stigma related to mental health treatment in younger adults.

### 4.2. Limitations and Future Recommendations

There are several limitations to the current study. First, the number of subjects used (n = 9 for the treatment group) and the duration of the treatment may not have been large enough or long enough to fully document the effects of the intervention. In addition, the current sample may not have been representative as many individuals with IGD also have comorbidities [132]. However, those participants with comorbidities were excluded due to a low number of participants that we were able to include in this study sample [98]. Additionally, the symptom severity in this sample was low (IGD ≤ 5); therefore, the findings must be considered only as preliminary evidence. The scale used to measure IGD (i.e., IGDT-10) has a Cronbach’s alpha under the generally accepted threshold (i.e., 0.68). Even though the scale has been widely used and validated in various languages, the lower validity may influence the selection of the treatment sample in our study. Therefore, the results should be taken as preliminary evidence. The scoring of the scale may also be a limitation. The scale treats only the severe presence of a criterion as an IGD symptom. This enables reducing pathologizing “healthy” gamers. But, at the same time, it also reduces the chance of individuals with less severe scores to receive attention for their problematic gaming. Ideally, future studies should use a clinical interview to confirm the presence of IGD symptomatology.

Another limitation is the lack of randomization in the gamer population. Due to a small portion of the targeted population being unwilling to participate in the study, volunteering participants were selected for the intervention group. This could be due to the fact that some gaming populations have a rare motivation to change [133]. It was important, however, that this study did not include strong extrinsic motivation such as payment as this may have acted as a form of contingency management and therefore compromised the variables related to gaming (e.g., mood or cognition). In addition, although the RGU group did not significantly differ on any of the important sociodemographic variables, there may have been other group differences that were not taken into consideration and could have influenced the results, such as personality factors or family relations.

Definite conclusions regarding the efficacy of a VR-based mindfulness intervention relative to other techniques can only occur within randomized controlled trials, which was not possible in this study due to a low number of volunteer participants that could be part of each group. Therefore, future studies would benefit from having well-controlled subjects with randomization, a larger sample size with more diversity, higher IGD symptom severity, and having long-term follow-ups to assess the potential mediators of change.

### 4.3. Clinical Implications

Despite its limitations, this study provides valuable inputs regarding the effectiveness of a VR-based mindfulness intervention for IGD. With the results of this study, mental health professionals may start implementing this cost- and time-effective intervention for the treatment and prevention of IGD.

The intervention may be used as a stand-alone treatment option, with possible variations in the number of sessions and duration of treatment, or as a supplement to other treatment approaches. As previously mentioned, the research shows that a combination of different treatments is usually the most effective approach when dealing with IGD symptomatology [23,24,25]. A VR-based intervention may be used in combination with classical pharmacotherapy, individual psychotherapy, or group therapy. Since it is cost-effective, schools and universities may utilize this approach for those who are at risk of an IGD diagnosis. Therefore, this intervention could possibly serve as a treatment, but also as a prevention technique.

With its consistency, this intervention could potentially serve as the first step towards a unified and standardized VR treatment for IGD.

In addition, the results may be used as an input for the development of a VR environment specifically suited for treating IGD. The findings from this study, combined with the findings of previous VR studies, could provide sufficient evidence and information for the development of an IGD-tailored VR intervention.

## 5. Conclusions

After a brief 4-week VR-based mindfulness intervention, the IGD symptoms and gaming time during the weekend were decreased in subjects who are at risk of IGD. We suggest that a VR-based intervention may improve the symptom severity and maladaptive gaming patterns in subjects who are at risk of an IGD diagnosis. Considering the significant reduction in symptoms, its simplicity, practicality of use, and cost- and time-effectiveness, a VR-based mindfulness intervention seems to be an effective and promising tool for the treatment of IGD.

## Figures and Tables

**Table 1:** Demographical and Internet-gaming characteristic measures of IGD and HC individuals at baseline.

Total n = 27	IGD (n = 9)	RGU (n = 10)	HC (n = 8)	
	Mean ± SD	Mean ± SD	Mean ± SD	*p* Value
Age	20.33 ± 1.12	21.50 ± 2.76	22.63 ± 2.50	0.138
IGD symptoms	2.22 ± 1.3	0.00	0.00	<0.001, IGD > RGU **, IGD > HC **
Weekday gaming time	2.56 ± 1.01	2.00 ± 1.56	0.00	<0.001, IGD > HC **, RGU > HC **
Weekend gaming time	3.56 ± 1.01	1.90 ± 0.87	0.00	<0.001, IGD > HC **, RGU > HC *
Daily online time	5.33 ± 1.12	4.8 ± 1.23	4.00 ± 1.51	0.155
PSS	23.78 ± 6.83	18.30 ± 6.78	19.25 ± 4.62	0.233
PANAS-P	29.11 ± 7.99	30.80 ± 5.61	28.99 ± 5.32	0.608
PANAS-N	32.33 ± 8.66	24.80 ± 8.08	22.12 ± 5.46	0.020, IGD > HC *

Note: IGD = subjects with Internet Gaming Disorder symptomatology; RGU = recreational game users; HC = healthy control subjects; weekday gaming time = hours spent gaming per day during the week; weekend gaming time = hours spent gaming per day during the weekend; daily online time = time spent on the Internet per day; PSS = Perceived Stress Scale score; PANAS-P = positive affect; PANAS-N = negative affect, * *p* < 0.05, ** *p* < 0.01.

**Table 2:** IGD symptoms measured at the baseline and second test.

Total n = 27	IGD (n = 9)	RGU (n = 10)	HC (n = 8)	Main Effect of Group	Main Effect of Time	Interaction of Group and Time
	Mean ± SD	Mean ± SD	Mean ± SD			
IGD baseline	2.22 ± 1.30	0.00 ± 0.00	0.00 ± 0.00	F(2, 24) = 22.82, *p* < 0.001	F(1, 24) = 4.68, *p* = 0.041	F(2, 24) = 7.77, *p* = 0.002
IGD post-test	0.89 ± 1.05	0.20 ± 0.42	0.00 ± 0.00			

Note: IGD = subjects with Internet Gaming Disorder symptomatology; RGU = recreational game users; HC = healthy control subjects; IGD baseline = IGD symptoms before the intervention/treatment time; IGD post-test = IGD symptoms after the intervention/treatment time.

**Table 3:** Wilcoxon signed rank test.

	IGD (n = 9)		
	Mean ± SD	z	*p*
IGD symptoms pre-treatment	2.22 ± 1.30		
IGD symptoms post-treatment	0.89 ± 1.05	−2.46	0.014
Weekday gaming pre-treatment	2.56 ± 1.01		
Weekday gaming post-treatment	2.22 ± 1.09	−0.966	0.334
Weekend gaming pre-treatment	3.56 ± 1.01		
Weekend gaming post-treatment	2.89 ± 1.45	−2.12	0.034

Note: IGD = subjects with Internet Gaming Disorder symptomatology; weekday gaming pre-/post-treatment = hours spent gaming per day during the week pre-/post-treatment; weekend gaming pre-/post-treatment = hours spent gaming per day during the weekend pre-/post-treatment.

## Data Availability

Data can be requested from the corresponding author.
